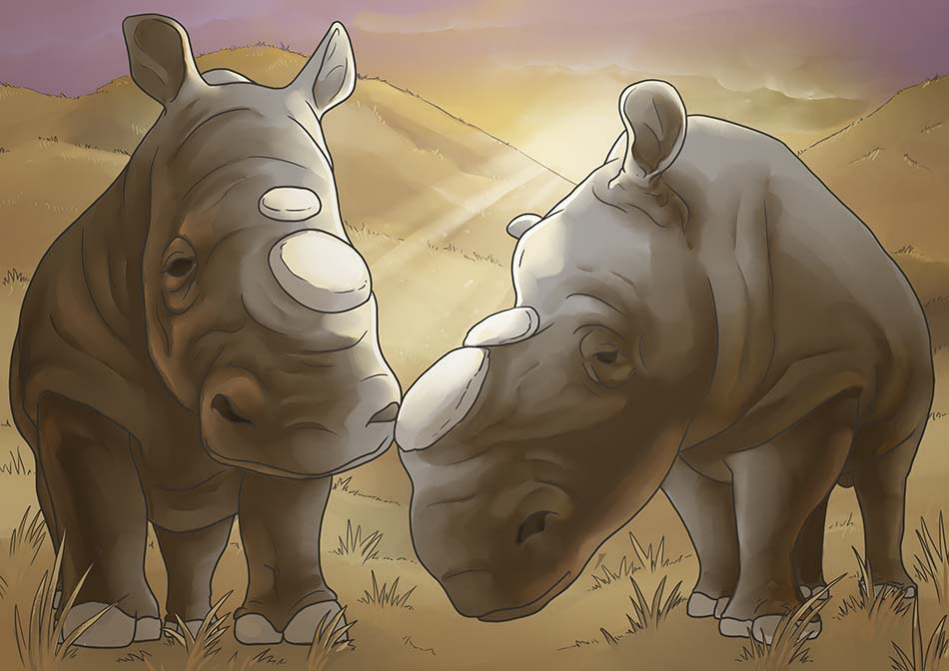# Horn today, gone tomorrow—dehorning as an anti-poaching practice for white rhinos

**DOI:** 10.1093/conphys/coab012

**Published:** 2021-03-29

**Authors:** Christine L Madliger

**Affiliations:** Carleton University, 1125 Colonel By Dr, Ottawa, Ontario K1S 5B6, Canada

Since the beginning of the 20th century, rhino populations have declined from 500 000 individuals to just 27 000. That means they are at just 5% of their population size since 1900! With increasing poaching pressure driven by a demand for rhino horn, the national parks and private reserves that are integral to the remaining rhinos’ survival often need on-the-ground security to protect them. But this type of protection can be expensive. As an alternative, some reserves in South Africa have turned to dehorning—a veterinary procedure that removes a portion of the rhino’shorn.

But should managers worry that dehorning causes stress that could impact the welfare of the rhinos?

This question is particularly pressing because when managers use dehorning as an anti-poaching tactic, they must trim the rhino’s horns every 1–3 years over its lifetime. Each time the procedure is performed, the rhino needs to be tranquilized. This repetition is necessary because the horns are made of keratin, the same material found in fingernails, claws and hooves. And just like those structures, rhino horns grow continuously.

Researchers at the University of Brighton investigated whether the dehorning procedure causes long-term stress for wild white rhinos by analysing hormones in their faeces before and after the procedures. They measured stress hormones called glucocorticoids, which have been shown to rise when rhinos are faced with stressors such as translocation or captivity. They also measured reproductive hormones, which tend to be suppressed under conditions of stress. If the physiology of the rhinos is impacted, it could mean they will face issues when they try to mate and produce offspring.

The rhinos in the study were darted from a helicopter and stabilized before having both of their horns trimmed, which is no easy task! The veterinary team must use a chainsaw to remove the horn material and then smooth the edges with a disc sander. They then reverse the effects of the anaesthetic to allow the rhinos to fully recover. This entire process takes about 15–20 minutes.

Penny *et al*. (2020) found that dehorning did not cause a long-term elevation in stress hormone levels compared with rhinos who had not undergone the procedure. They also showed that repeatedly dehorning over multiple years did not lead to increased stress hormone levels or the suppression of reproductive hormones in either male or female rhinos.

The researchers outline that further studies are needed to monitor the behaviour of rhinos after dehorning and to confirm that the procedure does not impact rhinos differently in other geographic areas or habitat types. Still, these results are encouraging. Dehorning is considered a relatively drastic measure, and so knowing that the rhinos’ physiology is not negatively affected by these procedures can help managers feel more confident about choosing it as an anti-poaching measure when the pressure to protect the rhinos’ safety is so high.

Illustration by Erin Walsh; Email: ewalsh.sci@gmail.com

Editor: Jodie L. Rummer